# Rare *RNF213* variants and the risk of intracranial artery stenosis/occlusion disease in Chinese population: a case-control study

**DOI:** 10.1186/s12881-019-0788-9

**Published:** 2019-03-29

**Authors:** Xin Liao, Tong Zhang, Bingyang Li, Shimin Hu, Junyu Liu, Jing Deng, Hongzhuan Tan, Junxia Yan

**Affiliations:** 1 Department of Epidemiology and Health Statistics, XiangYa School of Public Health, Central South University, Changsha, 410078 Hunan China; 2 Department of Neurology, the Second Hospital of Hebei Medical University, Heping West Road, Xinhua District, Shijiazhuang, 050000 Hebei China; 3 Department of Neurosurgery, XiangYa Hospital, Central South University, 87 Xiangya Road, Changsha, 410008 Hunan China

**Keywords:** *RNF213*, Intracranial artery stenosis, Rare variants

## Abstract

**Background:**

*RNF213* rare variant-p.R4810K (rs112735431) was significantly associated with intracranial artery stenosis/occlusion disease (ICASO) in Japan and Korea and to a lesser degree in China. Considering the allelic heterogeneity, we performed target exome sequencing of *RNF213* with the aim to identify the rare variants spectrum and their association with ICASO in a Chinese population and further to explore whether the rare variants carrier patients present specific clinical phenotype.

**Methods:**

Target exome sequencing of *RNF213* was performed in 250 ICASO patients using FastTarget sequencing technology. Various filtering process were used to select the candidate variants. Control individuals were obtain from 1000 Genome Project (208 Chinese samples) and GeneSky in-house database (1007 samples). Gene-based association analyses were conducted to identify the association between *RNF213* rare variants and ICASO. The clinical characteristics of rare variant carriers and non-carriers were compared using Chi-squared test or Fisher’s exact test.

**Results:**

After filtration, 18 rare variants were identified in 39 patients. Gene-based association test showed that rare variants of *RNF213* were significantly associated with ICASO (Minor allele frequency < 0.05, WSS *p* = 4.88 × 10^− 10^; SKAT *p* = 9.68 × 10^− 6^; SKAT-O *p* = 3.42 × 10^− 9^). There were no significant clinical characteristic differences other than the diagnosis age which was older in the carriers than the non-carriers (60.5 ± 6.2 vs 57.3 ± 8.9 years old, *p* = 0.028).

**Conclusion:**

Rare variants of *RNF213* are associated with ICASO in Chinese. However, there are limited genetic diagnosis values of the gene due to no specific phenotypic presentation in the carriers and non carrier patients.

**Electronic supplementary material:**

The online version of this article (10.1186/s12881-019-0788-9) contains supplementary material, which is available to authorized users.

## Background

Intracranial artery stenosis/occlusion disease (ICASO) is one of the main causes of ischemic stroke [1]. It is more prevalent in Asians than in Westerners. In Asia, 30–50% of strokes and more than 50% of transient ischemic attacks are caused by ICASO [2]. Atherosclerosis, chronic injury, infection, cardioembolism, arterial dissection, vasculitis, moyamoya disease (MMD) and rare autoimmune diseases are common cause of ICASO [3]. The most common cause of ICASO is atherosclerosis, which is commonly caused by acquired factors, such as smoking, hypertension, diabetes mellitus and dyslipidemia [3]. However, conventional risk factors only explain part of risks and the underlying etiologies is largely unknown. Many studies supported a genetic risk role of ICASO [3–10].

The ring finger protein 213 (*RNF213*) was found to be a susceptibility gene for MMD in East Asian population and the founder rare variant-p.R4810K (rs112735431) was subsequently identified to be associated with non-MMD ICASO which showed stenosis or occlusion of the intracranial major arteries but do not meet the diagnostic criteria of MMD [3–6, 11–21]. Meta-analysis showed that *RNF213* p.R4810K increased the risk of ICASO in Chinese (odds ratio, 5.59; 95% confidence interval, 2.12–14.75; *p* = 0.001), but the effect size was significantly lower than that in Japanese and Korean (odds ratios, 10.71 and 28.52; 95% confidence interval, 3.97–28.91; and 11.04–73.67, respectively) [22]. Further studies found that many non-p.R4810K *RNF213* rare variants have been identified in MMD patients in ethnically diverse populations, including Asians, whites, and Hispanics, while p.R4810K is absent in non-Asian populations, which illustrating allelic genetic heterogeneity of *RNF213* in different ethnic population [23]. Whether other *RNF213* rare variants contribute to Chinese ICASO is still unknown. Considering the allelic heterogeneity, we performed target exome sequencing of *RNF213* with the aim to identify the rare variants spectrum and their association with ICASO in a Chinese population and further to explore whether the rare variants carriers present specific clinical phenotype or not.

## Methods

### Study population

Seven hundred and fifteen ICASO patients were recruited from two university affiliated hospitals (615 from the Second Hospital of Hebei Medical University and 100 from XiangYa Hospital of Central South University) previously [9]. Among them, only 6 patients carried *RNF213* p.P4810K variants. In order to investigate whether other *RNF213* rare variants associated with ICASO or not, 250 patients were randomly selected from our previous individuals. Allelic frequencies of control individuals were obtained from 1000 Genome Project Chinese Han Population (208 samples, http://www.internationalgenome.org/home) and GeneSky in-house database (1007 samples, http://www.geneskybiotech.com/index.html). This study was approved by the Medical Ethics Committee of Central South University and all patients signed an informed consent before participating.

### Diagnosis of ICASO

The ICASO was diagnosed by digital subtraction angiography, computed tomography angiography or magnetic resonance angiography and defined as stenosis or occlusion at terminal and/or proximal portions of the intracranial major arteries without abnormal vascular networks in the basal ganglia that did not meet the diagnostic criteria of MMD. At least 2 physicians(1 radiologist and 1 neurological physician) were required to interpret angiography images results. Demographic information including age, gender, behavioral risk factors (tobacco and alcohol consumption), disease histories of hypertension, diabetes, hyperlipemia were collected by interview or questionnaire. Patients with potential diseases including cardioaortic embolism, coagulopathy, vasculitis, arterial dissection that may leads to cerebral apoplexy were excluded.

### FastTarget sequencing

Genomic DNA with high quality suitable for sequencing was extracted from the peripheral blood leukocytes using TIANamp Blood DNA Extraction Kit (TIANGEN BIOTECH CO., LTD, Beijing, China). Protein coding sequences of *RNF213* were selected and a custom panel covering the target regions was designed. Capture target regions contained flanking sequences of 25 bases at the borders of each exon. Target sequencing was performed on Illumina Hiseq/MiSeq high-throughput sequencing platform (Illumina, San Diego, CA, USA) according to the manufacturer’s instructions. Raw fastQ files were aligned to the human reference sequence (NCBI Genome build GRCh37, https://www.ncbi.nlm.nih.gov/genome/guide/human/#download) with the Burrows-Wheeler Aligner (BWA, http://bio-bwa.sourceforge.net/). The HaplotypeCaller from the Genome Analysis Toolkit (GATK, https://software.broadinstitute.org/gatk/best-practices/) and VarScan (http://varscan.sourceforge.net/) were used for variant calling in the target region. Quality of sequencing was determined with FastQC (http://www.bioinformatics.babraham.ac.uk/projects/fastqc/). PolyPhen-2 (http://genetics.bwh.harvard.edu/pph2/) and SIFT (http://sift.bii.a-star.edu.sg/) were used for predicting the deleterious level of the functional variants.

### Variant filtration

After sequencing, a series of filters were used to prioritize variants. Variants were considered higher priority if they were: (a) predicted to affect protein-coding sequences (including nonsynonymous, frameshift deletion, stop-gain variants in exonic or splicing regions); (b) less common in reference databases (Minor allele frequency was less than 0.05 in 1000 Genome project Chinese Han population [ftp://ftp.1000genomes.ebi.ac.uk/vol1/ftp/release/20130502/] and GeneSky in-house Database [http://www.geneskybiotech.com/]); (c) predicted as damaging, possibly damaging or unknown by SIFT or PolyPhen-2; (d) sequencing read depth was higher than 10x.

### Statistical analysis

Descriptive analyses were conducted using SPSS 21.0 software (SPSS Inc., Chicago, IL, USA). Continuous variable (age) was presented as the mean ± standard deviation (SD). Categorical variables (hypertension, diabetes, hyperlipemia, smoking, drinking, clinical symptoms and characteristics of stenosis) were presented as proportions. The Weight Sum Statistic (WSS), the Sequence Kernel Association Test (SKAT) and the SKAT-optimal test (SKAT-O) were used to evaluated the statistical significance of variants between affected and control individuals. A continuous weight function proposed by Madsen and Browning was performed to focus on the lower frequency and rarer variants, this weight score method allows both low frequency (MAF < 0.05) and rare (MAF < 0.01) variants to contribute to the overall statistic by calculating the logistic weights for each variant and applying them to the analysis according to the following formula [24]: *w*_*j*_ = 1/[*MAF*_*j*_(1 − *MAF*_*j*_)]^1/2^. Student’s t-test, Chi-squared test or Fisher’s exact test were used to compare the characteristic differences between carriers and non-carriers of *RNF213* rare variants. Threshold of statistical significance was set at *p* value less than 0.05.

## Result

### Characteristics of participants

Of all the 250 patients with ICASO, 77(30.8%) were female and the average age was 57.8 ± 8.6 years (range: 28–70 years). The information of the vascular risk factors, clinical symptoms and characteristics of cerebral artery stenosis were summarized in Table 1.

**Table 1:** Characteristics of the case participants and controls^d^

Characteristics	ICASO(*n* = 250)
Age(years)	
Mean ± SD [Range]	57.8 ± 8.6 [28–70]
Female*,* n(%)	77(30.8)
Risk factors, n(%)	
Smoking	97(38.8)
Drinking	63(25.2)
Hypertension	152(60.8)
Diabetes	60(24.0)
Hyperlipemia	95(38.0)
Clinical symptoms, n(%)	
Hemorrhage	7(2.8)
Infarction	165(66.0)
Ischemia	77(30.8)
Other	9(3.6)
Multiple stenosis	199(79.6)
Location	
Anterior circulation^a^	36(14.4)
Posterior circulation^b^	99(39.6)
Multiple circulation^c^	111(44.4)
Others	4(1.6)

^a^Anterior circulation = *ACA* Anterior cerebral artery, *MCA* Middle cerebral artery, *ICA* Internal carotid artery

^b^Posterior circulation = *BA* Basilar artery, *VA* Vertebral artery, *PCA* Posterior cerebral artery

^c^Multiple circulation = including both Anterior and posterior circulation

^d^Control individuals were obtained from 1000 Genome Project Chinese Han Population and GeneSky in-house database, clinical characteristics were not available

### Identification of low frequency and rare variants in target region

The average value of mean target coverage was 527x, 88.87% of the reads had a Phred-like quality score (Q-score) greater than 30.The proportion of the samples obtained mean coverage >140x was 94.24%. After variants filtration, 18 variants were found in 39 patients (Table 2). The detail information of these rare variants was shown in Table 3.

**Table 2:** Variant Filtration Steps of sequencing and results of gene-based analysis

Filtration Steps	Number of variants		
1.SNVs^a^ located in exonic/ splicing region	188		
2. Nonsynonymous SNVs/ frameshift deletion/ stopgain	125		
3. Variants judged as SIFT prediction = Damaging / unknown and PolyPhen-2 prediction = Possibly damaging/ Probably damaging/ Unknown	71		
4. MAF < 0.05 in 1KGP(Chinese Han population) and GeneSky in-house Database	42		
5.Variants with sequenced base depth > 10x	18		
Result of *RNF213* gene-based analysis			
MAF	P_WSS_	P_SKAT_	P_SKAT-O_
< 0.05	4.88 × 10^−10^	9.68 × 10^−6^	3.42 × 10^−9^
< 0.01	4.56 × 10^−12^	2.80 × 10^−6^	3.20 × 10^−11^

^a^*SNVs* Single Nucleotide Variants

**Table 3:** Rare variants detected in ICASO patients

Position	Gene Region	Function	Variant^a^		SNP ID	Genotype^b^			SIFT^c^	POLYPhen V2^c^
			cDNA	Amino Acid		Case	1000G_CHB	GENESKY		
Chr17:78247076–78,247,076	exonic	frameshift	c.134delC	p.S45 fs	–	249/1/0	208/0/0	1007/0/0	–	–
Chr17:78298891	exonic	missense	c.3086 T > C	p.L1029S	rs753208141	249/1/0	208/0/0	1007/0/0	D	D
Chr17:78307986	exonic	missense	c.4225G > T	p.D1409Y	–	249/1/0	208/0/0	1007/0/0	D	D
Chr17:78311532	splicing	–	c.4668 + 6C > T	–	rs78795452	248/2/0	205/3/0	1007/0/0	–	–
Chr17:78311620	splicing	–	c.4669-13A > G	–	rs750893752	249/1/0	208/0/0	999/8/0	–	–
Chr17:78313764	exonic	missense	c.5597C > T	p.T1866I	rs546687179	244/6/0	207/1/0	1007/0/0	D	–
Chr17:78318465	splicing	–	c.6343-13C > G	–	rs141121193	246/4/0	206/2/0	992/15/0	–	–
Chr17:78319385	exonic	missense	c.7250 T > G	p.I2417S	rs181965032	244/6/0	201/7/0	992/15/0	D	P
Chr17:78326772	exonic	missense	c.10336C > T	p.R3446W	rs776943470	249/1/0	208/0/0	992/15/0	D	D
Chr17:78350088	splicing	–	c.13186-13 T > C	–	rs113236556	239/11/0	202/6/0	1006/1/0	–	–
Chr17:78353469	exonic	missense	c.13595 T > C	p.I4532T	rs373648166	249/1/0	208/0/0	975/32/0	D	D
Chr17:78354738	exonic	missense	c.13748G > A	p.R4583Q	rs199887580	249/1/0	208/0/0	1007/0/0	D	D
Chr17:78355494	exonic	missense	c.13945C > G	p.L4649 V	rs61745599	249/1/0	208/0/0	1007/0/0	D	D
Chr17:78356830	exonic	missense	c.14030G > T	p.W4677 L	rs61741961	249/1/0	208/0/0	1006/1/0	D	D
Chr17:78357541	exonic	missense	c.14135A > T	p.N4712I	–	249/1/0	208/0/0	1007/0/0	D	D
Chr17:78360656	exonic	missense	c.14887C > T	p.R4963C	rs772035323	249/1/0	208/0/0	1007/0/0	D	D
Chr17:78362497	splicing	–	c.15000 + 8C > T	–	–	249/1/0	208/0/0	1006/1/0	–	–
Chr17:78363181	splicing	–	c.15195 + 14C > T	–	rs373144473	249/1/0	208/0/0	1007/0/0	–	–

^a^ Genbank accession number: NM_001256071; ^b^ Genotype presented as wild type/heterozygous/hommozygous

^c^ SIFT Score Prediction: D = damaging, T = tolerated; c POLYPHEN Score Prediction: B = benign, P = possibly damaging, D = probably damaging

– Not available

### Distribution of rare variants in ICASO patients and gene-based association test

Eighteen rare *RNF213* variants were found in ICASO including 6 splicing site variants, 11 missenses and 1 frameshift deletion. All the missense variants were predicted to be deleterious, 6 of them were absent from the control individuals, and 6 of them were clustered at the C-terminal domain of *RNF213* (exon 42–68) (Table 3, Additional file 1: Figure S1). We identified a total 42 alternative alleles in ICASO patients (*n* = 250) and 117 alternative alleles in controls (*n* = 1215, cumulative allele OR, 1.74; 95%CI, 1.23–2.49, *p* = 0.002). Single-variant association test revealed that none of the SNPs was associated with ICASO (Table 4). Therefore, we focus on gene-based burden test to evaluate the aggregate effects of low MAF variants. We performed WSS, SKAT, SKAT-O test at two MAF thresholds (MAF < 0.05, 0.01) respectively. The WSS and SKAT-O test using logistic weights to compare *RNF213* functional variants (MAF < 0.05) showed significantly different allelic distributions between the ICASO patients and control individuals (WSS *p* = 4.88 × 10^− 10^; SKAT *p* = 9.68 × 10^− 6^; SKAT-O *p* = 3.42 × 10^− 9^.). For the rarer *RNF213* functional variants (MAF < 0.01), gene-based test showed more significant difference (WSS *p* = 4.56 × 10^− 12^; SKAT *p* = 2.80 × 10^− 6^; SKAT-O *p* = 3.20 × 10^− 14^) (Table 2).

**Table 4:** Single variant test and cumulative allele odds ratio of *RNF213* rare variants

Variant	Ref	Alt	Case_Ref	Case_Alt	Control_Ref	Control_Alt	OR	95%CI	*p* value^*^
Chr17:78247076–78,247,076	C	–	499	1	2430	0	–	–	0.171
Chr17:78298891	T	C	499	1	2430	0	–	–	0.171
Chr17:78307986	G	T	499	1	2430	0	–	–	0.171
Chr17:78311532	C	T	498	2	2419	11	0.88	(0.20–4.00)	1.000
Chr17:78311620	A	G	499	1	2430	0	–	–	0.171
Chr17:78313764	C	T	494	6	2414	16	1.83	(0.71–4.71)	0.248
Chr17:78318465	C	G	496	4	2403	27	0.72	(0.25–2.06)	0.383
Chr17:78319385	T	G	494	6	2408	22	1.33	(0.54–3.30)	0.716
Chr17:78326772	C	T	499	1	2429	1	4.87	(0.30–77.95)	0.312
Chr17:78350088	T	C	489	11	2392	38	1.42	(0.72–2.79)	0.312
Chr17:78353469	T	C	499	1	2430	0	–	–	0.171
Chr17:78354738	G	A	499	1	2430	0	–	–	0.171
Chr17:78355494	C	G	499	1	2429	1	4.87	(0.30–77.95)	0.312
Chr17:78356830	G	T	499	1	2430	0	–	–	0.171
Chr17:78357541	A	T	499	1	2430	0	–	–	0.171
Chr17:78360656	C	T	499	1	2429	1	4.87	(0.30–77.95)	0.312
Chr17:78362497	C	T	499	1	2430	0	–	–	0.171
Chr17:78363181	C	T	499	1	2430	0	–	–	0.171
Total counts			8958	42	43,623	117	1.74	(1.23–2.49)	0.002

^*^P values were calculated using a two-sided Fisher’s exact test

*Ref* Reference allele(s), *Alt* Alternate allele(s)

Case = 250 ICASO patients, Control = 1KG Chinese Han population and GeneSky Database(including 1007 healthy individuals)

### Clinical characteristics of patients with and without *RNF213* rare variants

Among the 250 ICASO patients, we found 39 patients carried *RNF213* rare variants and compared the clinical characteristics between the carriers and non-carriers. The results showed that patients with *RNF213* rare variants were older at diagnosis (60.5 ± 6.2 vs 57.3 ± 8.9 years old, *p* = 0.028). Risk factors of ICASO (including smoking, alcohol drinking, hypertension, diabetes, hyperlipemia), clinical symptoms and characteristics of cerebral artery lesions (quantity of lesions, presence of anterior and posterior ICASO) showed no significant difference between two groups (Table 5).

**Table 5:** Characteristics of *RNF213* rare variants carriers and non-carriers

Characteristics	Carrier(*n* = 39)	Non-carrier(*n* = 211)	*p* value
Age(years)			
Mean ± SD	60.5 ± 6.2	57.3 ± 8.9	0.028
Female, n(%)	12(30.8)	65(30.8)	0.996
Risk factors, n(%)			
Smoking	16(41.0)	81(38.4)	0.756
Drinking	14(35.9)	49(23.2)	0.095
Hypertension	29(74.4)	123(58.3)	0.059
Diabetes	12(30.8)	48(22.7)	0.281
Hyperlipemia	14(35.9)	81(38.4)	0.768
Clinical symptoms, n(%)			
Hemorrhage	3(7.7)	4(1.9)	0.137
Infarction	29(74.4)	136(64.5)	0.23
Ischemia	8(20.5)	69(32.7)	0.13
Other	1(2.6)	8(3.8)	
Characteristics of cerebral artery stenosis, n(%)			
Multiple stenosis	29(74.4)	170(80.6)	0.377
Location			
Anterior circulation^a^	4(10.3)	32(15.2)	0.688
Posterior circulation^b^	14(35.9)	85(40.3)	
Multiple circulation^c^	20(51.3)	91(43.1)	
Others	1(2.6)	3(1.4)	

^a^Anterior circulation = *ACA* Anterior cerebral artery, *MCA* Middle cerebral artery, *ICA* Internal carotid artery

^b^Posterior circulation =*BA* Basilar artery, *VA* Vertebral artery, *PCA* Posterior cerebral artery

^c^Multiple circulation = including both Anterior and posterior circulation

## Discussion

In this study, we sequenced the exome of *RNF213* in 250 ICASO patients and identified 18 rare variants in 39 patients. Gene-based association test showed that rare variants of *RNF213* were significantly associated with ICASO. However, there were no significant clinical characteristic differences between the rare variants carriers and non-carriers.

*RNF213* founder mutation-p.R4810K (rs112735431) was identified to be significantly associated with MMD and non-MMD ICASO in East Asia. Other than p.R4810K, numerous rare variants also were identified in MMD cases in previous studies. In Japan, Kamada et al. identified 3 non-p.R4810K rare variants of *RNF213* (p.M3891 V, p.V4567 M and p.V4765 M) among MMD patients [11]. Subsequently, Moteki et al. found 16 rare coding variants of *RNF213* in MMD cases by exome sequencing, including 2 previously identified variants (p.V4567 M and p.V4765 M) and 14 novel variants (Additional file 1: Figure S1, Additional file 2: Table S1) [25]. In China, researchers identified 40 *RNF213* rare variants among MMD patients. All of these variants were specific in Chinese MMD except p.D4013N (found in Caucasian) and p.R4062Q (found in Caucasian and Japanese) [12, 14, 18, 21, 26]. Moreover, p.D4863N, p.E4950D and p.A5021V were particularly identified among Chinese MMD patients in several studies [12, 14, 21]. In non-East Asian countries, numerous *RNF213* variants were identified. Cecci et al. identified 10 variants only in Caucasian MMD patients [27] and Shoemaker et al. identified 22 variants in multiple non-East Asian population [26] (Listed in Additional file 2: Table S1). Whether these rare variants or other *RNF213* rare variants associated with ICASO like p.R4810K or not is unclear currently. In this study, we performed target exome sequencing of *RNF213* in ICASO patients and found 18 rare variants in 39 patients. All the 18 rare variants identified were novel and not have been found in previous MMD studies. This result suggested that *RNF213* rare variants spectrum of Chinese ICASO is distinct from MMD, locus heterogeneity of *RNF213* is highly indicated between ICASO and MMD.

Recent studies suggested that *RNF213* genotypes were associated with MMD phenotypes, numerous studies confirmed that the homozygous p.R4810K variant carriers present earlier onset age, severe symptoms and prognosis [13, 17, 28]. Zhang et al. demonstrated Chinese MMD patients with *RNF213* p.R4810K were younger at diagnosis than those without *RNF213* rare variants (25 vs 29 years old, *p* = 0.049), more ischemic cases and more likely to occur at posterior cerebral artery [21]. In addition, researchers also identified the association of *RNF213* variants and ICASO phenotypes. Bang et al. indicated that Korean ICASO patients with *RNF213* p.R4810K variant were younger at diagnosis than those without the variant (52.6 ± 9.6 vs 56.8 ± 12.7 years old, *p* = 0.027) [6]. But our study showed that mean age at diagnosis of *RNF213* rare variants carriers was older than that of non-carriers. Considering the complicated current medical status of China, it is difficult to confirm the precise onset age during the slow progression of ICASO unless the patients seek a medical treatment at the early stage of ICASO (such as recurrent headache, dizziness and transient ischemic attack). The majority of patients (66.6%) in our study suffered from severe acute cerebral infarction as initial clinical symptoms, so the age at diagnosis in our research may not be able to reflect the true onset age of ICASO.

In our study, the presence of anterior and posterior ICASO showed no significant difference between *RNF213* rare variants carriers and non-carriers. This result is consistent with Bang’s study. They reported that the presence of proximal carotid artery and posterior ICASO showed no significant difference between *RNF213* p.R4810K carriers and non-carriers [6]. But in Shinya’s study, all of 10 *RNF213* p.R4810K carriers suffered anterior ICASO and no one suffered posterior ICASO [10], they defined the posterior circulation as the basilar artery and vertebral artery, patients with posterior cerebral artery stenosis were not found in their case series. The difference of patient selection may explain the discrepancy.

ICASO is caused by environmental and genetic risk factors. Though some specific rare variants of *RNF213* were identified in Chinese ICASO patients, but there were no specific clinical characteristics of the *RNF213* rare variants carriers, which illustrating that rather than *RNF213* variants there may be other risk factors of ICASO in Chinese. Studies focus on multiple factors including genetic and environmental are needed.

The limitation of this study should be mentioned. First, the sample size of our study was relatively small and the strictly matched cerebrovascular disease-free controls were not available; the reference allele frequencies of rare variants in unaffected individuals were obtained from 1000 Genomes Project and Genesky in-house database. Second, atherosclerosis is the most common cause of ICASO, it usually caused by acquired factors, such as hypertension, diabetes mellitus, dyslipidemia, and smoking. However, these information were not available in the control databases, so we cannot take these environmental risk factors as covariate when we implemented the gene-based association test.

## Conclusion

Rare variants of *RNF213* are associated with ICASO in Chinese, but there were no specific clinical characteristics of the *RNF213* rare variants carriers. Genetic diagnosis values for this gene among Chinese ICASO patients are limited. Rather than *RNF213* variants there should be other risk factors of ICASO in Chinese. Studies focus on multiple factors including genetic and environmental are needed.

## Additional files

Additional file 1: **Figure S1.** *RNF213* variants identified in MMD and ICASO patients around the world. (TIF 712 kb)

Additional file 2: **Table S1.** Rare variants of *RNF213* identified in worldwide MMD patients. (XLSX 14 kb)

## Abbreviations

ACA: Anterior cerebral artery
BA: Basilar artery
BWA: Burrows-Wheeler Aligner
GATK: Genome Analysis Toolkit
ICA: Internal carotid artery
ICASO: Non-moyamoya intracranial major artery stenosis/occlusion
MAF: Minor allele Frequency
MCA: Middle cerebral artery
MMD: Moyamoya disease
PCA: Posterior cerebral artery
PolyPhen-2: Polymorphism Phenotyping v2
*RNF213*: The ring finger protein 213
SIFT: Sorting Intolerant From Tolerant
SKAT: Sequence Kernel Association Test
SKAT-O: SKAT-optimal test
VA: Vertebral artery
WSS: Weight Sum Statistic
