# Supplementary figures and images for: Rare RNF213 variants and the risk of intracranial artery stenosis/occlusion disease in Chinese population: a case-control study
Source: BMC Med Genet. 2019 Mar 29;20:55. doi: 10.1186/s12881-019-0788-9 (PMC6441181; doi:10.1186/s12881-019-0788-9)

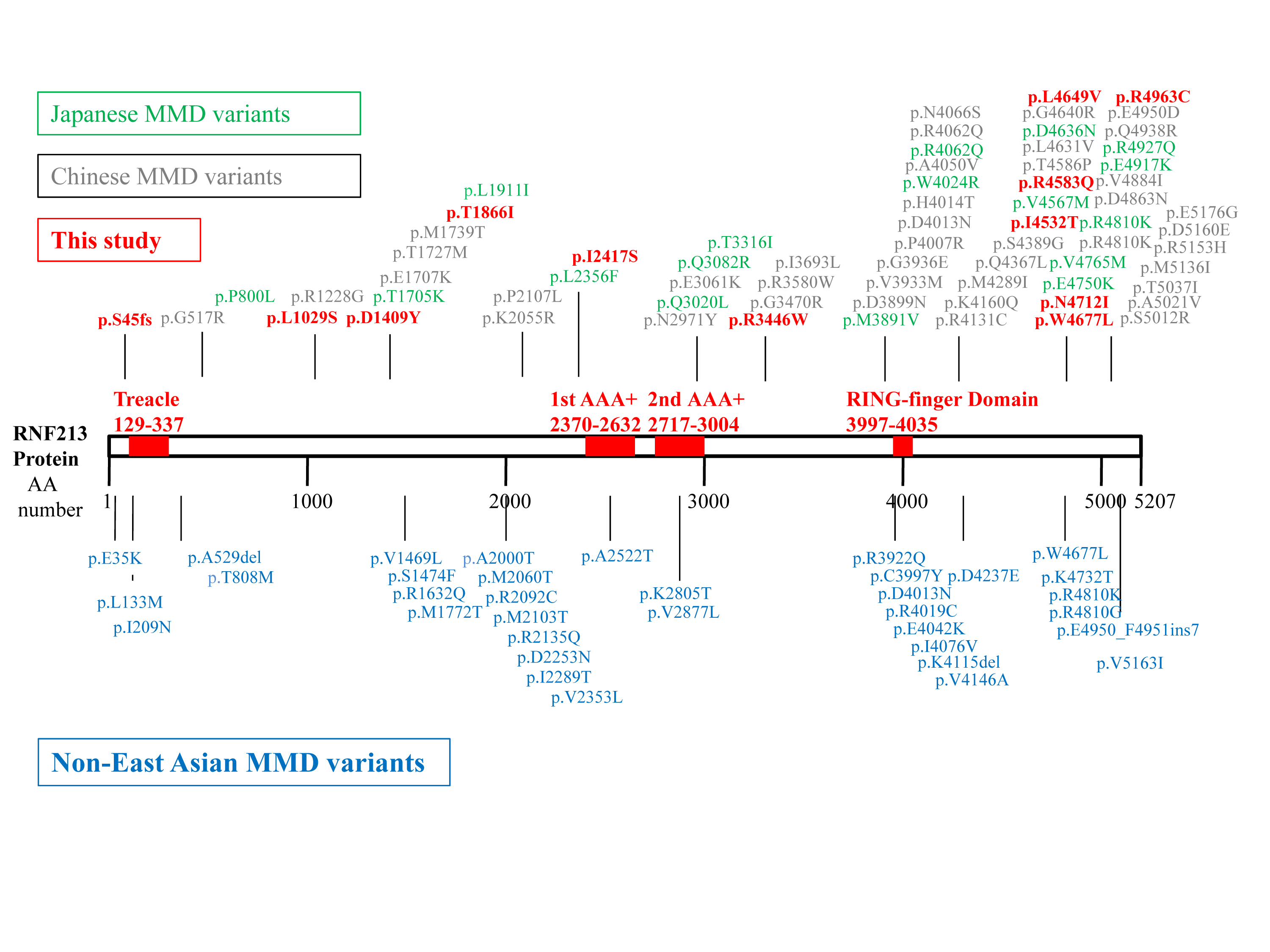

Supplement: Supplementary file 1 — Figure S1. RNF213 variants identified in MMD and ICASO patients around the world. (TIF 712 kb) [file 12881_2019_788_MOESM1_ESM.tif]
